# Association of *Helicobacter pylori* related chronic atrophic gastritis and gastric cancer risk: a literature review

**DOI:** 10.3389/fmed.2025.1504749

**Published:** 2025-02-20

**Authors:** Zefeng Zhang, Sitong Chen, Shudan Li, Yadan Zheng, Lifei Mai, Xiaoguang Zhang

**Affiliations:** ^1^Department of Digestive Endoscopy Center, Guangdong Provincial People's Hospital (Guangdong Academy of Medical Sciences), Southern Medical University, Guangzhou, Guangdong, China; ^2^Southern Medical University, Guangzhou, Guangdong, China

**Keywords:** chronic atrophic gastritis, gastric cancer risk, precancerous lesion, *Helicobacter pylori*, intestinal metaplasia

## Abstract

Chronic atrophic gastritis (CAG) is considered to be closely related to *Helicobacter pylori* (*H. pylori*) infection and characterized by the atrophy and/or intestinal metaplasia (IM) of the gastric mucosa in pathology. CAG is often regarded as the precancerous lesion of gastric cancer and *H. pylori* infection stimulates the development of atrophy and IM and the progression of gastric cancer through the persistent effect acting on the gastric mucosa, including releasing inflammatory factors such as Interleukin-8(IL-8). From the molecular biology perspective, growing evidence shows that *H. pylori* probably induce the expression of NF-κB, miR-204, miR-27a, hnRNPA2B1, and JARID1B, which play crucial roles in the progression of CAG into gastric cancer. In addition, *H. pylori* can increase Epstein-Barr virus (EBV) infection, and the co-infection will jointly increase gastric cancer risk. Furthermore, *H. pylori* induces cellular senescence and promotes atrophy progression and finally increases the gastric cancer risk. This review aims to explore the carcinogenic mechanisms of *H. pylori* related CAG in order to provide theoretical foundations for the pathogenesis mechanism and early detection and prevention of gastric cancer.

## Introduction

Chronic atrophic gastritis (CAG) is a disease characterized by chronic inflammation of the gastric mucosa, which typically presents a gradual atrophy of gastric glands and a reduction in gastric acid secretion (1–3). Based on clinical and etiological analysis, there are two major divisible types, that is, autoimmunity (usually appears in the corpus) and *Helicobacter pylori* (*H. pylori*) infection related (usually appears in the antrum) CAG. Autoimmune atrophic gastritis (AAG) was defined as including gastric body atrophy, no *H. pylori* present infection, negative *H. pylori* antibody in serum, and one of the three situations (positive intrinsic factor antibody findings, positive parietal cell antibody findings, Pepsinogens I ≤ 70 ng/mL and ratio of Pepsinogens I/Pepsinogens II ≤ 3). Although AAG also runs a certain risk of developing gastric cancer, the probability of malignant transformation is relatively lower compared to *H. pylori*. Interestingly, a portion of AAG patients are simultaneously appearing to have *H. pylori* infection; and sometimes AAG patients are misdiagnosed as refractory to *H pylori* eradication therapy, probably because achlorhydria might allow urease-positive bacteria other than *H pylori* to colonize the stomach, causing positive 13C-urea breath test results (4). Therefore, CAG caused by *H. pylori* infection will be the focus of discussion in this review.

*H. pylori* has been recognized as a Class I carcinogen (5) and plays a significant role in the pathogenesis of CAG (6, 7). The prolonged state of reduced protective barriers and low pH not only affects the normal function of the stomach but also significantly increases gastric cancer risk (8, 9).

Most gastric cancer cases are diagnosed at an advanced stage due to the lack of noticeable symptoms before then. Unfortunately, the median survival period for T3 or T4 stage gastric cancer without treatment is only 3–5 months, while those receiving palliative chemotherapy and radiotherapy have a median survival of 6–14 months (10). The small difference between these two scenarios underscores the poor prognosis of advanced gastric cancer; however, the 5-year survival rate can reach more than 90% for the early-stage (11). This highlights the importance of early diagnosis and treatment for gastric cancer patients. In this review, we tried to summarize the association between *H. pylori*-related chronic atrophic gastritis (CAG) and gastric cancer risk, focusing on mechanisms including intestinal metaplasia(IM), epigenetics, co-infections, and cellular senescence. We tried to help clinical physicians to better understand *H. pylori* related pathogenesis in CAG and gastric cancer risk, from all aspects (8–11). It will probably help to provide new methods to treat CAG and gastric cancer. Maybe it can inspire clinical physicians to explore new ideas to block the CAG progression after understanding the comprehensive pathogenesis.

## Intestinal metaplasia (IM)

IM is the state whereby the normal columnar epithelium of the stomach is substituted with intestinal epithelium and is considered as a precancerous lesion. IM is often diagnosed using magnifying endoscopy (ME) and endoscopic biopsies, which is important in diagnosis of CAG and gastric cancer and during follow-up. IM frequently arises in chronic gastritis, especially after *H. pylori* infection; while individuals with autoimmune-related CAG predominantly exhibit glandular atrophy without signs of IM (12). This implies that *H. pylori* may make gastric mucosa adopt intestinal characteristics after infection during the course of CAG.

Several mechanisms have been described to explain how *H. pylori* induces chronic inflammation in gastric mucosa, such as the well-known cytotoxin-associated gene A (CagA), vacuolating cytotoxin A (VacA), and the urease alpha subunit (UreA). Gastric mucosal cells take up outer membrane vesicles (OMVs) containing toxins through endocytosis or phagocytosis, and CagA and VacA play an important role in the process as pathogenic factors (13). Autophagy is treated as a self-defense machinery that fights against *H. pylori* infection to inhibit DNA-damage accumulation and the proliferation of precancerous gastric cells. However, CagA and VacA have been reported to disrupt the process of autophagy through down-regulation of human autophagy gene Beclin1(BECN1) (14). This suppression of autophagy further leads to mitochondrial damage, accumulated receptor protein SQSTM1/p62 autophagy substrate, increased release of reactive oxygen species (ROS), and finally promoted inflammation and oxidative stress (15). In this way, CagA impairs intracellular signaling and induces Interleukin-8(IL-8) secretion of gastric epithelial cells in a phosphorylation-independent manner. The level of IL-8 markedly influences the degree of mucosal inflammation, which thus promote gastric mucosa inflammation (16). It has also been presented the mRNA expression level of Recombinant Enolase 1 (ENO1) in chronic gastritis and precancerous lesions in the *H. pylori* infection group were obviously higher than in the non-infection group. The same results of ENO1 expression difference were obtained with cancerous and adjacent tissues in gastric cancer. Thus, ENO1 can be considered as a response to *H. pylori* infection and CagA transfection, showing that *H. pylori* positive gastric mucosal is under stimulation after infection (17).

In terms of immunological perspective, CagA increases the expression of programmed death ligand-1 (PD-L1) in gastric cancer-cell-derived exosomes by inhibiting p53 and miRNA-34a, which inhibits proliferation and anticancer effects induced by CD8^+^T cells (18). Tissue-resident memory T (TRM) cells generated by CD8^+^T cells develop aberrant T cell receptor beta chains during chronic infection, which results in the failure to maintain the TRM phenotype and eventually replaced by CD4^+^T cells to occupy the niche of CD8^+^T cells (19). In the meantime, the level of chemokine CCL3 and CX3CL1 in gastric mucosa infected with CagA^−^ *H. pylori* were much higher than those infected with CagA^+^ *H. pylori*, which more effectively promotes CX3CR1^+^CD4^+^ term cells recruitment in mouse gastric mucosa (20). This means that different *H. pylori* strains may induce several mechanisms to evade host immune defense, leading to persistent infection and development of chronic inflammation in gastric mucosa.

There is, in addition, one further point to make. In previous studies, it has been pointed out the virulence level of *H. pylori* depends on and is positively related to the types and quantities of virulence gene expression. Some scholars believe that there is no need to blindly take eradication treatment for those who are negative for CagA and VacA and have no clinical symptoms. Based on it, the application of gene editing technology (CRISPR-Cas12a) combined with recombinase polymerase amplification (RPA) provides a new technical means for the detection and virulence gene typing of *H. pylori* in clinical practice.

In Correa's cascade model, the progression from IM to cancer unfolds in a stepwise manner (20) and *H. pylori* infection is considered as a primary factor in initiating inflammatory responses (21). Under the interaction of chronic inflammation, *H. pylori* infection and IM, gastric stem cells may incur genetic damage resulting in irreversible genetic alterations. Research indicates that changes at both the genetic and epigenetic levels can drive IM toward low-grade dysplasia and/or high-grade dysplasia. It is estimated that ~20% of cases with low-grade dysplasia may progress to high-grade dysplasia (22, 23). Notably, in some instances, IM can be partially reversed following the eradication of *H. pylori* infection (24). However, when the condition advances to high-grade dysplasia, the associated risk of developing gastric cancer escalates dramatically. Existing studies have shown that individuals diagnosed with high-grade dysplasia get a 40-fold increase in their likelihood of progressing to gastric cancer compared to those without this condition (25).

**Figure 1:**
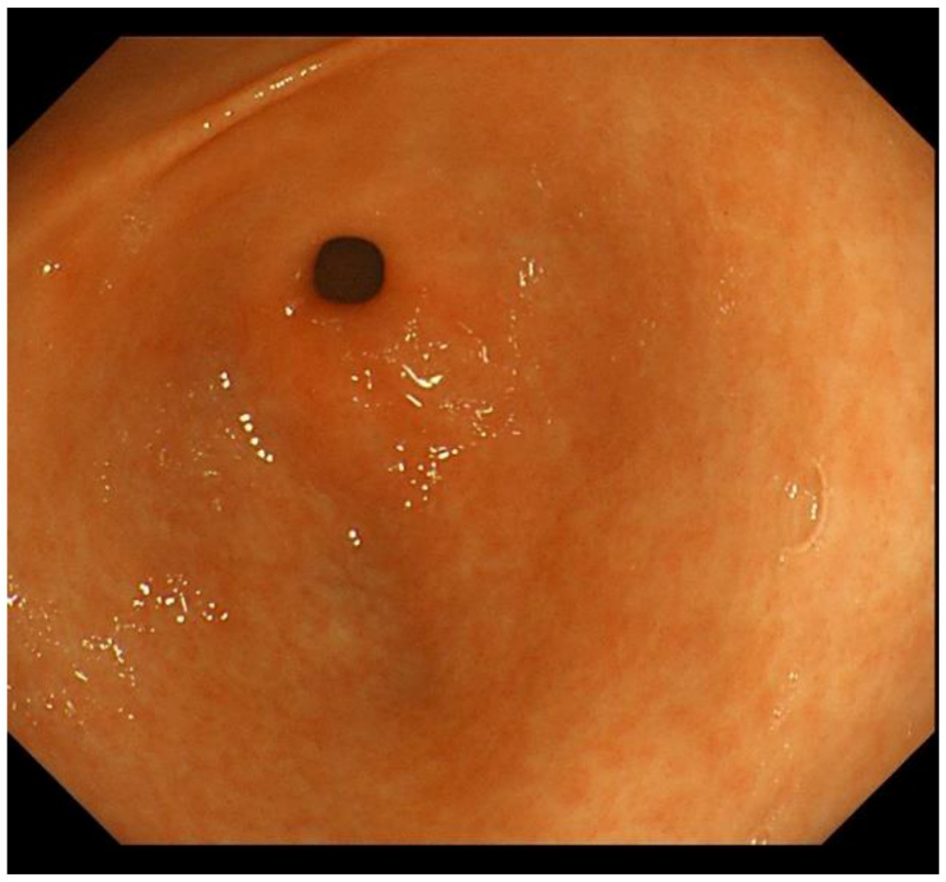
Normal gastric mucosa in antrum.

**Figure 2:**
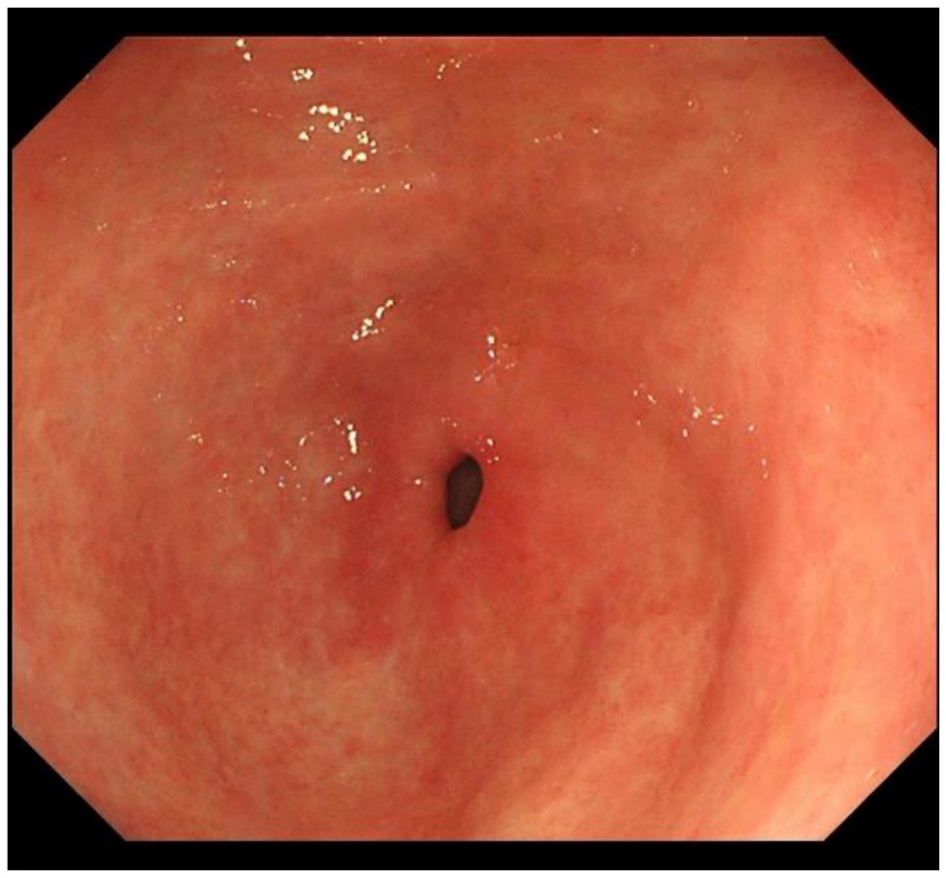
Atrophic gastric mucosa in antrum.

**Figure 3:**
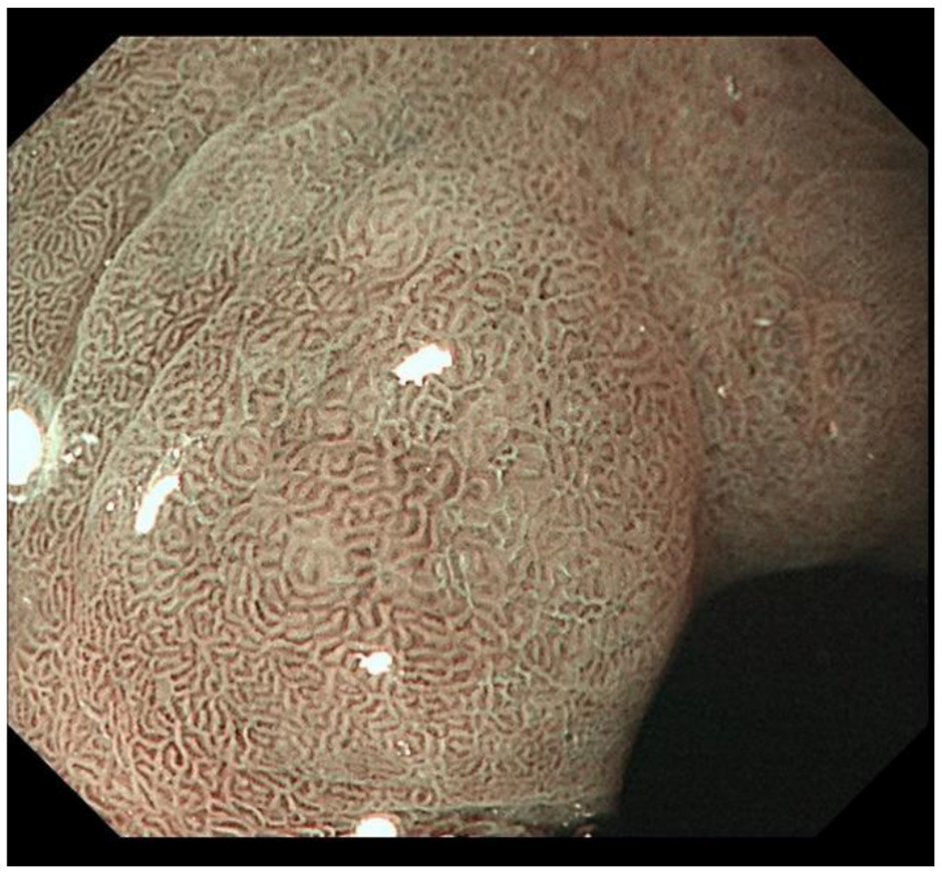
Typical intestinal metaplasia, where ME reveals structures on the mucosal surface with bright blue crests; IM gradually presents in the foveolar and glandular epithelium of antrum.

**Figure 4:**
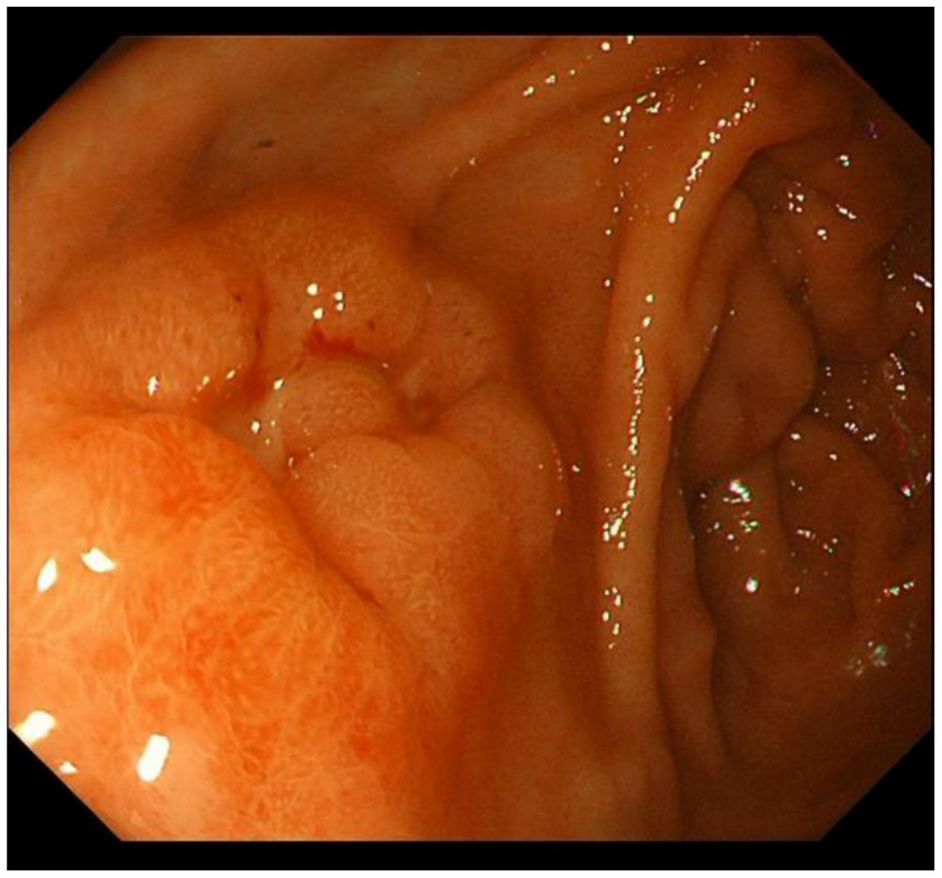
Early gastric cancer in antrum.

**Figure 5:**
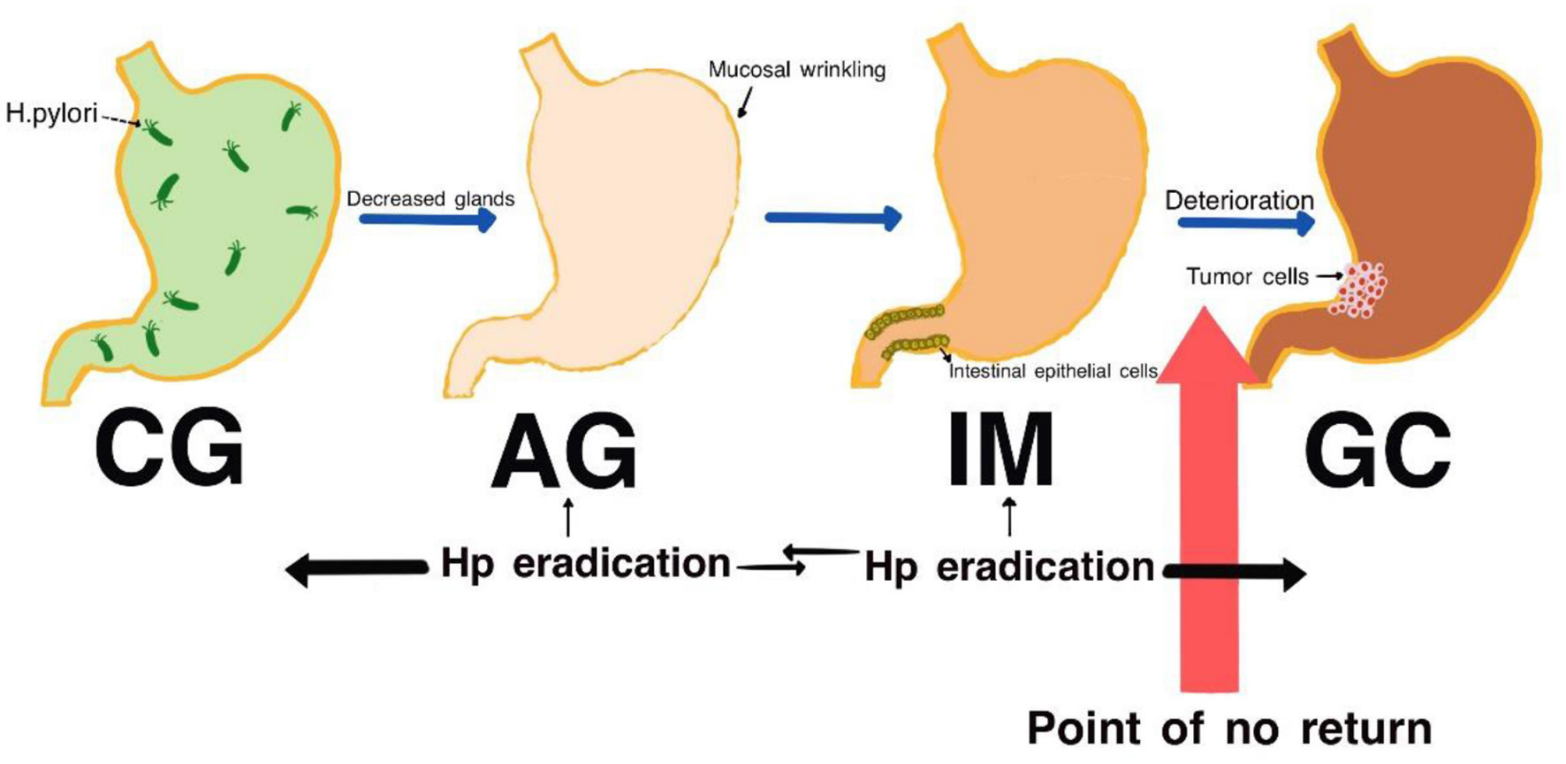
Correa cascade process (genetic mutations gradually accumulate in the stomach's epithelial stem cells until they reach a point of no return, after which they progress toward gastric cancer. The effectiveness of eradicating *H. pylori* diminishes as the cascade progresses).

IM typically originates from the antrum and generally spreads along the lesser curvature toward the fundus and corpus of the stomach as the condition advances (as the gastric shape line) (26). IM typically does not cause any symptoms and is usually discovered by ME and biopsies. Images below show different stages from normal mucosa, atrophic mucosa, IM and cancer in antrum by endoscopy (seen as Figures 1–4). Throughout this intricate process, various genetic and epigenetic alterations gradually accumulate over time, creating a complex web of changes. As these modifications continue to build up, they eventually lead to irreversible changes within the cellular structure and function. This accumulation of changes ultimately sets the stage for the development of gastric cancer. It has also indicated that metaplastic glands form through glandular fission and may possess similar genetic traits to aberrantly proliferating glands (27). This finding further supports the idea that genetic changes accumulate progressively before events leading to abnormal proliferation take place (26). The extent of involvement by IM correlates positively with the increasing gastric cancer risk (28). *H. pylori* elimination before IM presents appears to be an effective way to reduce gastric cancer risk. *H. pylori* eradication in early stage before notable changes in the gastric mucosa can help prevent more serious complications, particularly the progression to gastric cancer (29). Even if established IM cannot be reversed, eradicating *H. pylori* can still slow down the progression according to Correa's cascade model (30) (seen as Figure 5).

It is worth noting that bile reflux, irregular diets and taking painkillers (like NSAIDs) can also lead to IM, besides *H. pylori*. In clinical, we not only focus on *H. pylori* related IM, but should take note of the other factors. A detailed history is necessary to the cause diagnosis.

Operative Link for Gastritis Assessment (OLGA), firstly proposed in 2005, provided more intuitive information for clinical physicians to predict disease progression and formulate disease management measures, after linking the histopathology of chronic gastritis with the risk of cancer. Later, in 2010, Operative Link on Gastric Intestinal Metaplasia Assessment (OLGIM) was suggested due to the consistency of IM diagnosed by pathologists was much higher than atrophy. OLGA and OLGIM were complementary to each other and predicted the cancer risk in high risk areas of gastric cancer. High OLGA/OLGIM staging (stages III/IV) is an independent risk factor for the development of gastric cancer. OLGA/OLGIM has been promoted and widely accepted in Europe, Japan and China. Regular endoscopic examination and *H. pylori* eradication are strongly recommended for stage III/IV of OLGA/OLGIM.

## Epigenetic regulation

It is widely known that *H. pylori* infection is a significant cause of CAG and gastric cancer, while its direct role in triggering tumor-associated gene mutations may not be as prominent. Instead, recent research increasingly suggests that the development of gastric cancer is more closely linked to epigenetic abnormalities. These abnormalities can alter gene expression without changing the DNA sequence, leading to the activation or silencing of critical genes involved in cancer progression.

The general genetic background involved in gastric cancer is caused not by simple gene mutations, but by disruptions in DNA methylation patterns. This aberrant DNA methylation results in silencing cell cycle regulatory genes, the tumor suppressor gene p53 signaling pathway, and even the activation of some oncogenic signaling pathways, such as the WNT pathway, to promote the occurrence and development of gastric cancer (31). Christodoulidis has demonstrated that *H. pylori* infection might affect the DNA methylation patterns of the host cells through mediating its pathogenic factor, CagA protein. It was further identified that such abnormal methylation change induced by CagA might cause the silencing and inactivation of several key tumor suppressor genes. These methylations suppress the expression of normal genes, hence promoting the progression of gastric cancer (32).

**Figure 6:**
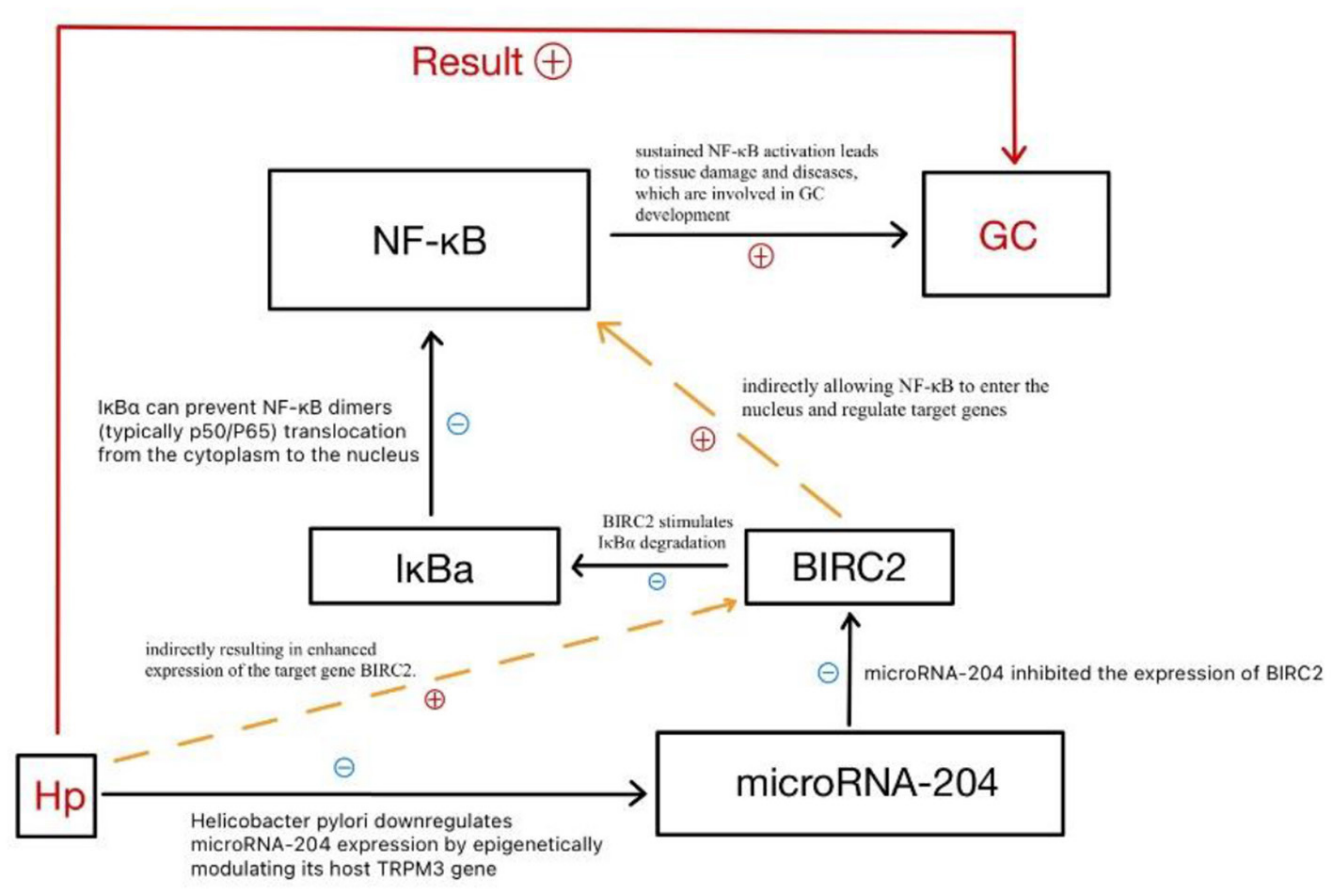
*H. pylori* increases gastric cancer risk through NF-κB pathway.

Utilizing the GEO dataset GSE28700, Chen identified a total of 16 abnormally expressed microRNAs in gastric cancer tissues as compared with adjacent normal tissues. Downregulation of microRNA-204 expression showed the most obvious difference according to their result. This was further confirmed in another study of IgG (+) gastric cancer patients, in which microRNA-204 was also observed to be significantly lower in gastric cancer tissues. Overexpression of microRNA-204 may inhibit the migration and invasion of gastric cancer cells and suppress potential lung metastatic. Moreover, *H. pylori* infection and/or its pathogenic factor CagA may induce downregulation of microRNA-204 expression in human gastric epithelial cell line GES-1. Further analysis showed that the expression of microRNA-204 has been downregulated from normal gastric mucosa to chronic gastritis and then to gastric cancer. It was also found that the infection with *H. pylori* epigenetically regulates the expression of microRNA-204, thus promoting the activation of NF-κB signaling by increasing the expression of BIRC2 in GC and gastric epithelial cells. NF-κB pathway is proved to be critical in both innate and adaptive immune response. Continuous activation of NF-κB often leads to pathologic tissue damages and diseases, which are related to the development of gastric cancer (33) (seen as Figure 6).

Besides, microRNA-204 can modulate gastric cancer development due to its regulatory effect on the NF-κB signaling pathway by influencing tumor microenvironment features including angiogenesis and immune responses. Other than BIRC2, microRNA-204 works as a tumor suppressor in regulating several target genes in a variety of cancers. It also downregulates the anti-apoptotic gene BCL-2 in gastric cancer cells, enhancing their sensitivity to chemotherapy agents like 5-fluorouracil (5-FU) and oxaliplatin, which are closely related to clinical prognosis (34). Downregulation of microRNA-204 increases the activities of target genes such as BDNF, Sox4 and EphB2, and promotes the migration and stem cell-like phenotype of glioma cells (35). In conclusion, microRNA-204 represents one of the main regulatory factors in the course of gastric cancer induced by *H. pylori* infection, and has provided new targets for the prevention and treatment of gastric cancer.

Besides that, *H. pylori* infection not only induced activation of the NF-κB signaling pathway, but also recruited NF-κB to the promoter region of the hnRNPA2B1 gene and caused its upregulation in gastric cancer cells. Subsequently, the overexpression of hnRNPA2B1 increased its interaction with PABPC1-eIF4F complex and led to following regulation of protein synthesis, promotion of mRNA levels and malignant progression in gastric cancer. It should be noted that the process is independent of m6A modification, although m6A plays an important role in mRNA metabolism; this is mediated by hnRNPA2B1, an m6A reader protein that delivers m6A-dependent mRNA stabilization in cancer (36). While hnRNPA2B1 can lower the glycolytic metabolism in gastric cancer cells, inhibit tumor growth and reduce liver metastasis, it also makes these cells more sensitive to the chemotherapy drug cisplatin (CDDP). This means that hnRNPA2B1 might play dule roles in the intricate web of gastric cancer regulation. Overall, *H. pylori* infection tends to push the progression of gastric cancer into a more aggressive phase by initiating NF-κB signaling pathway and promoting the expression of hnRNPA2B1. HnRNPA2B1 drives tumor malignancy and plays a crucial role in regulating the transcription, translation, and other processes of important oncogenes. In gastric cancer, the high expression of hnRNPA2B1 is closely related to poor prognosis in patients, mediating the process of malignant transformation such as gastric cancer migration and invasion. XI-101, as novel small molecule ligand of hnRNPA2B1, disruptes the binding of hnRNPA2B1 to the MDMX promoter and UTR and inhibits MDMX transcription, upregulates of p53, enhances expression of proapoptotic genes as downstream of p53, and ultimately induces apoptosis of gastric cancer cells. The Chemical targeting of hnRNPA2B1-MDMX-p53 axis has certain significance for the treatment of gastric cancer. These findings offer new perspectives on the molecular mechanisms behind the onset and progression of gastric cancer, opening up novel potential treatments (36).

Although *H. pylori* infection is a major contributor to gastric cancer, it also works by changing the epigenetic characteristics of host cells. This alteration can affect the expression of key oncogenes, which play a crucial role in cancer development. Knowing well this mechanism provides new insights to explore into the molecular processes behind gastric cancer. It also offers chances for developing targeted therapies that focus on epigenetic regulation.

## Co-infection with Epstein-Barr virus (EBV)

EBV infection accounts for ~10% of total gastric cancer cases in the world, roughly 1.3 and 20.1% (37). Infection with *H. pylori* facilitates EBV infection by inducing the expression of auxiliary EBV receptor EphA2 and NMHC-IIA and their interaction in gastric epithelial cells. Cell surface localization of EphA2 is critical for EBV infection. The exposure of *H. pylori* not only increases the expression of EphA2, but also helps the formation of EphA2 and NMHC-IIA complex, which together enhance EBV infection. Apart from that, the capability of *H. pylori* infection to promote EBV infection is also attributed to its cag pathogenicity island instead of specific virulence factors such as CagA, VacA or FlaA. These findings suggest chronic *H. pylori* infection might lead to EBV-associated gastric cancer (38).

Co-infection with EBV and *H. pylori* was reported to increase gastric cancer risk by 3.3 times in patients (39). Co-infection with these two pathogens may have a synergistic effect on inducing inflammation of the gastric tissues and increase the development of gastric cancer. It is believed that gene product interactions are oncogenic, especially in the presence of antigens expressed from cytotoxin-related genes of *H. polyri*. On the other hand, Jin Hee Noh found that co-infection with EBV and *H. polyri* did not present a significant predictor concerning tumor biological outcome, suggesting that co-infection with EBV and *H. polyri* may be associated with an increased gastric cancer risk but probably not influence the clinical outcome and prognosis of patients (40).

Dharmendra indicated that co-infection created a favorable microenvironment which promoted high expression of pathogen-associated genes. The EBV latent genes, both ebna1 and ebna3c, had higher expressions in co-infection compared to the infection with EBV alone at 12 and 24 h. Simultaneously, *H. pylori* related genes, such as 16S rRNA, cagA and babA, were also found markedly upregulated in the course of co-infection. Gankyrin is known as a small oncoprotein and regulates different cellular signaling pathways during tumor progression. Interestingly, knockdown of Gankyrin was found to reduce drastically the cancer properties of gastric epithelial cells with an expression pattern similar to ebna3c. *H. pylori* induce gastric cancer progression in infected gastric epithelial cells, interfere with the cell cycle, gastric cancer markers, cell migration, DNA response, and anti-apoptotic genes. This new study may offer a perspective to understand the interaction of these two oncogenic factors and their mechanism for enhancing the oncogenic activity of gastric epithelial cells through Gankyrin (41).

Based on the above foundation, Charu Sonkar performed experiments using the AGS and found that *H. pylori* infection significantly up-regulate the expression of ITK. Meanwhile, co-infection with EBV and *H. pylori* up-regulated EPHB6 and tyrosine protein kinase Fyn expression obviously. It was also demonstrated that co-infection of EBV with *H. pylori* enhanced gastric cancer cell proliferation and changes in cell morphology and survival through expression regulation of certain kinases and apoptosis-related genes. These observations may offer a clue to understand the mechanism of early gastric cancer progression. It is worth mentioning that, the variability induced by different strains of *H. pylori* has showed variable effects on cell proliferation. Accordingly, *H. pylori* does not seem to promote cell growth consistently and presents as a time-dependent and strain-dependent relationship (42).

## Cell senescence

Besides several well-known mechanisms, Cai has come up with a new pathway in which cellular senescence plays an important role in transforming CAG into gastric cancer due to the infection of *H. pylori* (43). The available literatures indicated that *H. pylori* can induce cellular senescence by activating p21 through its cytotoxin-associated gene A, which is known as CagA (44). Although cell senescence is generally considered as an absolute barrier to tumorigenesis, the aged cells would secrete many proteins and create a distinct “senescence-associated secretory phenotype (SASP).” The SASP includes proinflammatory cytokines and chemokines that elicit inflammatory responses with beneficial and detrimental consequences. For instance, the SASP may lead to immune suppression and promote the growth and invasion of tumor cells (45–47). The secretory phenotype can, therefore, activate the many inflammatory signals and transduction events, thereby facilitating cell proliferation, growth recurrence, and metastasis of tumors (48). The presence of aging cells might provide a valuable biomarker for assessing gastric cancer risk in patients with *H. pylori* related CAG and serve as the basis for endoscopic monitoring. By detecting the presence of aging cells in different precancerous lesions, Cai pointed that the distribution of aging cells was mainly concentrated in the atrophic area of the gastric mucosa. Moreover, they concluded that cellular senescence is a mechanism of *H. pylori* related CAG, and *H. pylori* promoted gastric epithelial cell senescence through C-X-C motif chemokine receptor 2(CXCR 2) signaling. It has been proved that blocking CXCR2 in mucosa infected with *H. pylori* in mice model can delay the development of precancerous gastric lesions. Therefore, they have summarized that treatment with CXCR2 inhibitors in the *H. pylori* infection at early stage could dramatically minimize neutrophil infiltration, cellular senescence, and mucosal atrophy, which can delay gastric cancer progression (43).

Recently, interventions targeting cellular senescence have gained more and more attention, especially after considering that *H. pylori* eradication has shown no apparent improvement in gastric atrophy in some cases. This may be because the bacteria can induce cellular senescence even in the absence of an active infection by upregulation of cytokines, such as tumor necrosis factor-alpha and interleukin-1 beta, which may induce through NFKB1 to activate CXCR2 signaling (49). Moreover, some of the virulence factors of *H. pylori* may be related to cellular senescence induction. According to the available literature, *H. pylori* strains with a functional type IV secretion system and CagA exhibit a higher virulence, inducing remarkable immune responses, which is regarded associated with the development of gastric cancer (44). It follows that CagA could induce gastric carcinogenesis by facilitating senescence of the gastric mucosa, but this is yet to be supported by hard evidence.

In a word, cell senescence plays a critical role in the process of canceration from *H. pylori* related CAG to precancerous lesions and then finally to gastric cancer (50). This phenomenon may have an important clinical implication, although further investigation and studies are still needed to make it clear.

## *H. pylori* eradication

Eradicating *H. pylori* is crucial for gastrointestinal diseases like dyspepsia, gastritis, duodenitis, peptic ulcers, G-MALT lymphoma, and gastric adenocarcinoma. CAG patients with Hp positivity should receive eradication treatment. Nowadays, the first-line *H. pylori* eradication is proton pump inhibitor and colloidal bismuth plus two antibiotics for 10–14 days at home and abroad. High bacterial load, high gastric acidity, *H. pylori* strain, smoking, low compliance, overweight, and increasing antibiotic resistance are regarded as treatment failure factors (51).

Recent studies have revealed that vonoprazan-based triple therapy is to be a highly effective first-line regimen (52). It should be emphasized that carbon-13 urea breath test or gastroscopy supervision are still recommended for response evaluation after *H. pylori* eradication, while serology alone may not be valid for the assessment of the efficacy of eradication treatment (53).

## Conclusion

Driven by *H. pylori* infection, IM progresses into gastric cancer under conditions of chronic inflammation, low pH, and other factors. Genetic mutations increase the risk of high-grade dysplasia and cancer, marking a critical step in Correa's cascade toward an irreversible pathway of gastric cancer development.

It is now widely accepted that *H. pylori* may promote gastric carcinogenesis through abnormal epigenetic changes in the host cells. Indeed, increasing evidence has supported the notion that gastric carcinogenesis has a closer relation with epigenetic rather than genetic alterations. *H. pylori* and its virulence factor as CagA up-regulate or suppress targeted gene expression, thereby improve gastric cancer risk. The discovery proposes new insights into the multifaceted molecular processes underlying gastric cancer development and also offers new epigenetic-based therapeutic strategies in gastric cancer.

*H. pylori* can increase opportunities for secondary EBV infection since the gastric epithelial cells begin to display extra EBV receptors like EphA2 and NMHC-IIA. This co-interaction implies us that *H. pylori* infection increases the odds of EBV positive gastric cancer and other EBV related cancers in host. Co-infection by *H. pylori* and EBV makes severe inflammation inside gastric tissues and finally improve gastric cancer risk. *H. pylori* and EBV, serving as pathogen-linked genes in cancerous conditions and gastric cancer, help to demonstrate the importance to know the impact of co-infection on gastric health.

In addition, cellular senescence is also newly discovered during the development of *H. pylori* related CAG into gastric cancer. *H. pylori* promotes gastric epithelial cell senescence through the activation of p21 and CXCR 2. Even with past infection of *H. pylori*, it still may trigger CXCR2 signaling to induce cellular senescence through upregulating the related cytokines like NFKB1. SASP originated from senescent cells promotes cell proliferation, tumor growth, recurrence and metastasis.

CAG significantly increases gastric cancer risk and should be taken seriously by clinicians. The progression from CAG to gastric cancer involves complex mechanisms, and future research still needed to explore deeper its mechanism, considering epidemiological variations among different ethnicities and regions. For high-risk individuals with CAG, early detection and regular follow-ups are valuable. Timely eradication should be initiated when *H. pylori* infection is confirmed; which not only reduces the incidence of gastric cancer, but also greatly enhances patient outcomes.
