# Corrigendum: Association of *Helicobacter pylori* related chronic atrophic gastritis and gastric cancer risk: a literature review

**DOI:** 10.3389/fmed.2025.1586952

**Published:** 2025-03-20

**Authors:** Zefeng Zhang, Sitong Chen, Shudan Li, Yadan Zheng, Lifei Mai, Xiaoguang Zhang

**Affiliations:** ^1^Department of Digestive Endoscopy Center, Guangdong Provincial People's Hospital (Guangdong Academy of Medical Sciences), Southern Medical University, Guangzhou, Guangdong, China; ^2^Southern Medical University, Guangzhou, Guangdong, China

**Keywords:** chronic atrophic gastritis, gastric cancer risk, precancerous lesion, *Helicobacter pylori*, intestinal metaplasia

In the published article, there was an error in the Funding statement. “The author(s) declare financial support was received for the research, authorship, and/or publication of this article. This study was supported by Guangdong Provincial People's Hospital Research Fund (8200100073/8210101308/8220102641/8230102561).” The correct Funding statement appears below.

